# Differential capacity of human interleukin-4 and interferon-α monocyte-derived dendritic cells for cross-presentation of free versus cell-associated antigen

**DOI:** 10.1007/s00262-015-1741-1

**Published:** 2015-07-28

**Authors:** Jurjen M. Ruben, Hetty J. Bontkes, Theresia M. Westers, Erik Hooijberg, Gert J. Ossenkoppele, Tanja D. de Gruijl, Arjan A. van de Loosdrecht

**Affiliations:** 1 Department of Hematology, Cancer Center Amsterdam, VU University Medical Center, De Boelelaan 1117, 1081HV Amsterdam, The Netherlands; 2 Department of Pathology, Cancer Center Amsterdam, VU University Medical Center, De Boelelaan 1117, 1081HV Amsterdam, The Netherlands; 3 Department of Medical Oncology, Cancer Center Amsterdam, VU University Medical Center, De Boelelaan 1117, 1081HV Amsterdam, The Netherlands

**Keywords:** Monocyte-derived dendritic cell, IL-4, IFNα, Antigen presentation, Vaccination

## Abstract

Dendritic cells (DC) vaccination is a potent therapeutic approach for inducing tumor-directed immunity, but challenges remain. One of the particular interest is the induction of an immune response targeting multiple (unknown) tumor-associated antigens (TAA), which requires a polyvalent source of TAA. Previously, we described the preferred use of apoptotic cell-derived blebs over the larger apoptotic cell remnants, as a source of TAA for both in situ loading of skin-resident DC and in vitro loading of monocyte-derived DC (MoDC). Recent reports suggest that MoDC cultured in the presence of GM-CSF supplemented with IFNα (IFNα MoDC), as compared to IL-4 (IL-4 MoDC), have an increased capacity to cross-present antigen to CD8^+^ T cells. As culture conditions, maturation methods and antigen sources differ between the conducted studies, we analyzed the functional differences between IL-4 MoDC and IFNα MoDC, loaded with blebs, in a head-to-head comparison using commonly used protocols. Our data show that both MoDC types are potent (cross-) primers of CD8^+^ T cells. Whereas IFNα MoDC were more potent in their capacity to cross-present a 25-mer MART-1 synthetic long peptide (SLP) to a MART-1_aa26-35_ recognizing CD8^+^ T cell line, IL-4 MoDC proved more potent cross-primers of antigen-specific CD8^+^ T cells when loaded with blebs. The latter is likely due to the observed greater capacity of IL-4 MoDC to ingest apoptotic blebs. In conclusion, our data indicate the use of IFNα MoDC over IL-4 MoDC in the context of DC vaccination with SLP, whereas IL-4 MoDC are preferred for vaccination with bleb-derived antigens.

## Introduction

Targeting residual leukemic cells using dendritic cell (DC) vaccination is a promising therapeutic strategy for the treatment of acute myeloid leukemia (AML), as well as other types of cancer. Since the use of DC was first proposed for active anti-tumor vaccination by Banchereau and Steinman [1], strategies for loading tumor-associated antigen (TAA) onto DC have been exploited intensively (reviewed for AML in [2, 3]). Loading DC with TAA is an essential step in inducing tumor-directed T cell responses, and the use of different antigenic sources has proven to affect DC function [2, 4–6], as well as the number of tumor antigen epitopes. Different strategies to load DC with TAA for anti-tumor T cell priming depend on different (intra)cellular routes of antigen processing. One of the most important capacities of DC in the context of antigen loading is their ability to ingest and cross-present exogenous antigen, a mechanism exploited to load DC with a variety of TAA sources for vaccination purposes. Exogenous antigens [e.g., from apoptotic cells or synthetic long peptides (SLP)] are most efficiently presented in complex with HLA class II [7]. However, DC can present exogenous TAA in HLA class I molecules by means of cross-presentation [8]. The exact mechanism by which cross-presentation occurs and which DC types are most potent in their ability to cross-present antigen in vivo remains unclear (reviewed in [9]). However, most DC subsets are capable of performing this task and appear to achieve this with similar efficacy [10]. Previously, we have reported that apoptotic blebs, derived from apoptotic AML cells, are a superior source of TAA for the in situ loading of human skin-resident DC, which take up and cross-present TAA from blebs to a higher degree as compared to ACR [11]. Moreover, IL-4 MoDC loaded with isolated apoptotic blebs increased CD4^+^ T cell proliferation and T helper (T_H_) type I skewing (based on IFNγ production), as compared to using ACR; importantly, we have also shown that bleb-loaded IL-4 MoDC induce TAA-specific CD8^+^ T cells with higher avidity [6].

Monocytes are commonly differentiated into MoDC in the presence of GM-CSF and IL-4. Currently, there is growing interest in the use of MoDC differentiated in the presence of GM-CSF and interferon-α (IFNα), since IFNα MoDC have been described to be more potent in cross-presenting antigen [12–14], which would be beneficial for DC-based tumor vaccines. However, the culture and maturation methods, as well as the antigen sources used in these studies vary. For instance, the culture periods used for the generation of both types of MoDC generally differ, i.e., a 3-day culture period for IFNα MoDC and a 5-day culture period for IL-4 MoDC. In order to synchronize the cultures from the same donors, most studies either increased the IFNα MoDC culture time or shortened IL-4 MoDC differentiation accordingly. Moreover, varying modalities have been used to induce DC maturation in these comparative studies, e.g., TNFα, lipopolysaccharides or CD40 ligation [12, 13], as well as different sources of antigen, e.g, soluble protein and apoptotic cells [12–14].

To determine the preferred DC type to serve as a vehicle for AML-derived apoptotic bleb vaccines, we conducted a head-to-head comparison of IL-4 MoDC and IFNα MoDC. These MoDC types were cultured using the commonly used culture protocols (5- vs. 3-day, respectively), matured with a maturation cocktail consisting of IL-1β, IL-6, TNFα, PGE-2, and subsequently analyzed for their immunophenotype, endocytic capacity for apoptotic blebs and soluble antigen, and their ability to prime naive T cells, to cross-present, and to cross-prime antigen-specific T cells.

## Materials and methods

### Generation of apoptotic cell fractions

In order to facilitate assessment of antigen-specific CD8^+^ T cell activation and induction, HL60 AML cells were retrovirally transduced with melanoma-associated antigen recognized by T cells (MART-1), as described previously [15]. Apoptotic cell fractions were generated as described previously [6]. In short, apoptosis was induced by leaving the HL60 cells for 2 h at 42 °C in serum-free RPMI (Gibco, Paisley, UK), followed by γ-irradiation at 5000 rad. After 48–72 h, ACR were removed from the preparation by centrifuging at 600×*g*, after which the resulting supernatant was spun down at 4000×*g* to isolate the apoptotic blebs. Next, the blebs were washed with PBS, and the protein concentration was determined using a ND-1000 Nanodrop spectrophotometer (Thermo Fisher Scientific, Breda, the Netherlands). The isolated blebs were stored in liquid nitrogen until use.

### Dendritic cell culture

Monocytes were isolated from peripheral blood mononuclear cells (PBMC) of healthy donors, after informed consent, by magnetically activated cell sorting using CD14 Microbeads (Miltenyi Biotec, Utrecht, the Netherlands). Isolated monocytes were cultured in the presence of 800 U/ml GM-CSF (Peprotech, the Netherlands), supplemented with either 500 U/ml IL-4 (Peprotech, the Netherlands) for the generation of IL-4 MoDC, or 1000 U/ml IFNα A/D (R&D Systems) for the induction of IFNα MoDC. IL-4 MoDC were cultured for 5 days and IFNα MoDC for 3 days, as most frequently described in literature [12–14, 16].

### Dendritic cell immunophenotype, cytokine production, and loading

After differentiation, MoDC were isolated and the immunophenotype was determined by flow cytometry, using FITC-labeled, PE-labeled, APC, Horizon V450, or PeCy7-labeled antibodies against HLA-ABC, HLA-DR, CD1a, CD14, CD36, CD40, CD80, CD83, CD86, CLEC9a, Lox-1, CD18/CD11b (complement receptor 3), and CD18/CD11c (complement receptor 4) (all obtained from BD Biosciences), and the expression levels were subsequently analysed using flow cytometry (LSRFortessa™, BD Biosciences); the data were analyzed using FACS Diva software (BD Biosciences). MoDC cytokine production was analyzed, after overnight co-culture with irradiated CD40 ligand-expressing J558 cells and 1000 U/ml IFNγ (Sanquin, Amsterdam, the Netherlands), using an inflammatory cytokine bead array (BD Biosciences, Breda, the Netherlands). For MoDC loading, 2 × 10^5^ MoDC were loaded with 40 µg of blebs in the presence of the differentiation cytokine cocktails (GM-CSF/IL-4, or GM-CSF/IFNα), and 1 h after initiating loading, maturation was induced by IL-1β (10 ng/ml), TNFα (200 U/ml, both from Sanquin, Amsterdam, the Netherlands), IL-6 (10 ng/ml, R&D systems, Abingdon, UK), and PGE2 (10 ng/ml, Sigma-Aldrich, Zwijndrecht, the Netherlands).

In order to determine uptake, 40 μg blebs were labeled with 0.5 μM carboxyfluorescein succinimidyl ester (CFSE) (Invitrogen, Breda, the Netherlands), and cultured overnight with 2 × 0^5^ PKH26 red-labeled (1 μM, Sigma-Aldrich) MoDC. The percentage of double-positive cells was analyzed using flow cytometry (LSRFortessa™), as a measure of uptake.

Endocytosis of soluble proteins was analyzed by adding either dextran-FITC (2 μg/ml, Sigma-Aldrich) or Lucifer Yellow (2 μg/ml, Sigma-Aldrich) to immature IL-4 or IFNα MoDC for 1 h, after which the uptake was analyzed using flow cytometry (LSRFortessa™).

### Mixed leukocyte reaction

Peripheral blood lymphocytes (PBL) were isolated after informed consent from PBMC of healthy donors, by depleting CD14^+^ cells using CD14 Microbeads (Miltenyi Biotec). PBL were stored in liquid nitrogen until further use. PBL were labeled with 1 µM CFSE (Invitrogen) and plated in a 96-well plate at 1 × 10^5^ per well. Mature bleb-loaded MoDC were added to the wells at DC/PBL ratios of 1:5, 1:10, or 1:20, and CD3^+^CD4^+^ and CD3^+^CD8^+^ T cell proliferations were analyzed using flow cytometry after 6 days. The day 6 supernatant was analyzed for T cell cytokines, using a TH1/TH2/TH17 cytokine bead array (BD Biosciences, Breda, the Netherlands).

### Antigen cross-presentation

HLA-A2^+^ MoDC were loaded with different concentrations of a 25-mer MART-1_aa16-40L_ SLP for 2 h, after which MoDC maturation was induced by adding IL-1β, IL-6, TNFα, and PGE-2. Blebs were loaded as described above, at 40 µg per 2 × 10^5^ MoDC. After loading SLP or blebs overnight, MoDC were harvested and co-cultured for 5 h with a MART-1_aa25-36_ recognizing CD8^+^ T cell line (MART-1 T cell line, >95 % pure), in the presence of 1 μl/ml GolgiStop™ (BD Biosciences). Next, the cells were washed and stained with an APC-labeled MART-1_aa26-35_ HLA-A2 dextramer (Immudex, Denmark) for 15 min, followed by 15 min of staining with Horizon V450 or FITC-labeled CD3 and CD8 mAbs (both BD Biosciences). After washing, the cells were fixed and permeabilized using BD Cytofix/Cytoperm™ solution (BD Biosciences), following manufacturer’s protocol. Finally, intracellular IFNγ (BD Biosciences) was stained for 30 min at 4 °C, after which the cells were washed and IFNγ production was analyzed using flow cytometry (LSRFortessa™, BD Biosciences), as a measure of activation.

### CD8^+^ T cell priming

CD14^+^ from HLA-A2^+^ PBMC were differentiated into either IL-4 MoDC (5 days) or IFNα MoDC (3 days), and CD8^+^ and CD14CD8^−^ cells were stored in liquid nitrogen for further use. IFNα MoDC or IL-4 MoDC were loaded with blebs or MART-1_aa26-35L_ peptide, and plated at 1 × 10^5^ per well. Autologous CD8^+^ T cells and irradiated CD14^−^CD8^−^cells were added to the loaded MoDC at 1 × 10^6^ per well, supplemented with 20 U/ml IL-7, 10 ng/ml IL-12 (both Sanquin), and 10 ng/ml IL-6 (R&D systems) in a final volume of 2 ml in Yssel’s medium [17]. After 24 h, 10 ng/ml IL-10 (eBioscience, Vienna, Austria) was added to the co-cultures. The priming cultures were restimulated after 9 or 11 days, and subsequently every 7 days, with freshly loaded (thawed) IFNα MoDC or IL-4 MoDC, respectively. The percentage of dextramer-positive cells was analyzed before each restimulation.

### T cell avidity assay

JY cells were loaded for 3 h with MART-1_aa26-35L_ peptide in serum-free RPMI, at concentrations ranging from 10 μM to 1 pM, in the presence of 3 μg/ml β2-microglobulin. 5 × 10^5^ CD3^+^CD8^+^T cells were cultured with 5 × 10^5^ peptide loaded JY, in the presence of 1 μl/ml GolgiStop™ (BD Biosciences) for 5 h, after which intracellular IFNγ production was assessed as described above.

### Statistical analysis

Statistical analysis was performed with GraphPad Prism version 5 for Windows (GraphPad Software Inc.), using a paired two-tailed Student’s *t* test, Fisher’s exact test, or a Wilcoxon matched-pairs signed-rank test as indicated. Differences with *p* values ≤0.05 were regarded significant.

## Results

### Differences in MoDC phenotype post-differentiation and maturation

**Fig. 1:**
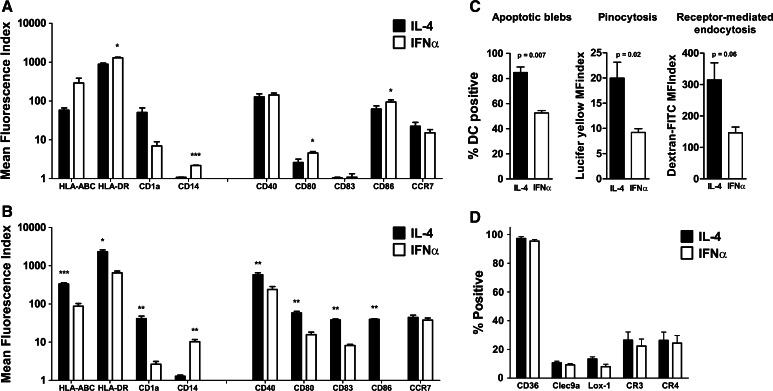
Analysis of MoDC immunophenotype and endocytic capacity. **a** Immunophenotype of monocyte-derived dendritic cells (MoDC) generated in the presence of either GM-CSF and IL-4 (*black bars*), or GM-CSF and IFNα (*white bars*), for, respectively, 5 or 3 days, (*n* = 4). *p < 0.05, **p ≤ 0.01, ***p ≤ 0.001. **b** Immunophenotype of MoDC 48 h after loading with blebs and subsequent maturation induction by IL-1β, IL-6, TNFα, and PGE-2 (*n* = 4). *p < 0.05, **p ≤ 0.01, ***p ≤ 0.001. **c** Fluorescently labeled MoDC were co-cultured overnight with fluorescently labeled blebs, after which the percentage of double-positive IL-4 (*black bars*), or IFNα MoDC (*white bars*), were quantified (apoptotic blebs). Alternatively, MoDC were cultured in the presence of Lucifer Yellow (pinocytosis), or dextran-FITC (receptor-mediated endocytosis). (*n* = 4). **d** Expression levels of scavenger receptors on immature IL-4 (*black bars*), or IFNα MoDC (*white bars*), using flow cytometry (*n* = 4). Statistical significance was for all figures determined using a Student’s *t* test

We first assessed MoDC phenotype after a 3- or 5-day differentiation period for IFNα or IL-4 MoDC, respectively (Fig. 1a). IFNα MoDC had significantly higher expression levels of HLA class II (*p* = 0.04), and CD14 (*p* = 0.0005), as compared to IL-4 MoDC. Moreover, IFNα MoDC showed a higher expression of CD80 (*p* = 0.01) and CD86 (*p* = 0.03). Next, we assessed the MoDC phenotype upon maturation induction in the presence of apoptotic blebs. In contrast to the immature state, IL-4 MoDC displayed a significantly higher expression of both HLA class I (*p* = 0.0006) and HLA class II (*p* = 0.01) and CD1a (*p* = 0.009) following loading with blebs and subsequent maturation (Fig. 1b). Moreover, IL-4 MoDC had a significantly higher expression of CD40 (*p* = 0.003), CD80 (*p* = 0.002), CD83 (*p* = 0.001), and CD86 (*p* = 0.002), whereas IFNα MoDC retained a higher CD14 expression after maturation (*p* = 0.009). After inducing maturation, IFNα MoDC down-regulated CD1a, compared to their immature expression levels (*p* = 0.02), at levels lower than those on mature IL-4 MoDC (*p* = 0.002).

### IL-4 MoDC are more potent in their ability to ingest blebs and soluble antigen

As the ingestion of exogenous antigen by MoDC is essential for the (cross-) priming of antigen-specific T cells, we analyzed the capacity of IFNα and IL-4 MoDC to phagocytose apoptotic blebs. After overnight co-culture, a significantly higher percentage of IL-4 MoDC had ingested blebs as compared to IFNα MoDC, in an immature (85 vs. 53 %, *p* = 0.0007; Fig. 1c, apoptotic blebs) as well as a mature state (75 vs*., 55* %, *p* = 0.025, data not shown). In order to determine whether this difference in the capacity to ingest blebs could be explained by different expression levels of receptors involved in antigen uptake and cross-presentation, we analyzed the expression levels of CD36, Clec9a, Lox-1, complement receptors (CR)3, and CR4 on IFNα versus IL-4 MoDC (Fig. 1d). We observed similar levels of expression of the tested receptors on both immature (Fig. 1d) and mature IFNα and IL-4 MoDC (data not shown). Lectin receptors are described to play a role in apoptotic cell uptake [18], and to assess whether IL-4 MoDC had an increased pinocytosis and lectin receptor-mediated antigen uptake, we performed a 1 h co-culture with either Lucifer Yellow (Fig. 1c, pinocytosis) or dextran-FITC (Fig. 1c, receptor-mediated endocytosis), respectively. Next, to an increased uptake of blebs, we observed an increased uptake of both Lucifer Yellow (*p* = 0.02) and a near-significant increase in the uptake of dextran-FITC (*p* = 0.06) by IL-4 MoDC, as compared to IFNα MoDC.

### Cytokine release and allogeneic T cell induction

**Fig. 2:**
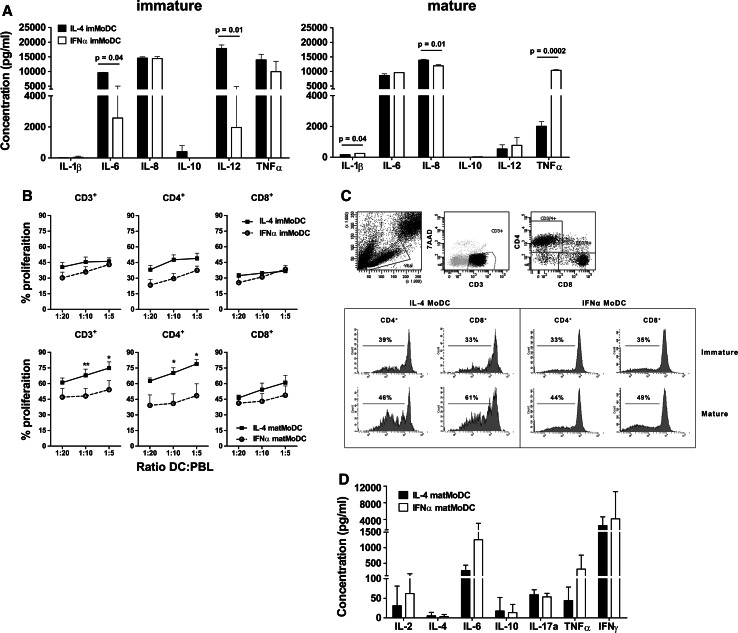
Cytokine production and T cell stimulation by MoDC. **a** Immature MoDC (left graph), or MoDC matured overnight using IL-1β, IL-6, TNFα, and PGE-2 (i.e., cytokine maturation cocktail, *right graph*), were ligated with CD40 in the presence of IFNγ, after which the produced cytokines were analyzed (*n* = 4). **b** MoDC were loaded with blebs in the absence (imMoDC) or presence (matMoDC) of the cytokine maturation cocktail, and subsequently co-cultured with fluorescently labeled allogeneic PBL in a mixed leukocyte reaction (MLR). After 6 days, CD4^+^ and CD8^+^ T cell proliferation was analyzed using flow cytometry. **c** The gating strategy and representative example of CFSE dilution following a MLR. **d** The produced T cell cytokines were analyzed in the supernatant (*n* = 4). Statistical significance was for all figures determined using a Student’s *t* test

IFNα or IL-4 MoDC were cultured overnight in the presence of IFNγ and the CD40 ligand-expressing cell line J558, after which the supernatant was analyzed for inflammatory cytokines. Immature IL-4 MoDC produced significantly higher amounts of IL-6 (*p* = 0.04) and IL-12p70 (*p* = 0.01) (Fig. 2a, immature) as compared to IFNα MoDC. No differences were observed in the secreted levels of IL-1β, IL-8, IL-10, and TNFα, after CD40 ligation of immature MoDC (Fig. 2a, immature). CD40 ligation following MoDC maturation led to a small but significant increase in the production of IL-8 by IL-4 MoDC, compared to IFNα MoDC. Mature IFNα MoDC produced marginally higher levels of IL-1β and considerably higher levels of TNFα, compared to IL-4 MoDC (Fig. 2a, mature).

To determine the potency of IFNα or IL-4 MoDC, loaded with blebs, to prime T cells, we co-cultured fluorescently labeled allogeneic PBL with bleb-loaded mature MoDC at different ratios. After 6 days of co-culture, we analyzed the induced T cell proliferation (Fig. 2b, c), and analyzed the produced T cell cytokines. Mature IL-4 MoDC induced a significant increase in CD3^+^ T cell proliferation over mature IFNα MoDC, whereas no significant differences could be detected between immature IFNα and IL-4 MoDC (Fig. 2b). The increase in T cell proliferation by mature IL-4 MoDC was attributable to the CD4^+^ T cell compartment, since no clear differences in proliferation could be detected within CD8^+^ T cell compartment (Fig. 2b). Analysis of the cytokines (IL-2, IL-4, IL-6, IL-10, IL-17a, TNFα, and IFNγ) produced by T cells after a 6-day co-culture with matured MoDC showed that despite a difference in proliferation, no significant differences were induced in the released T cell cytokines between the IFNα and IL-4 MoDC co-cultures (Fig. 2d).

### IFNα MoDC have an increased capacity to cross-present a MART-1 synthetic long peptide, compared to IL-4 MoDC

**Fig. 3:**
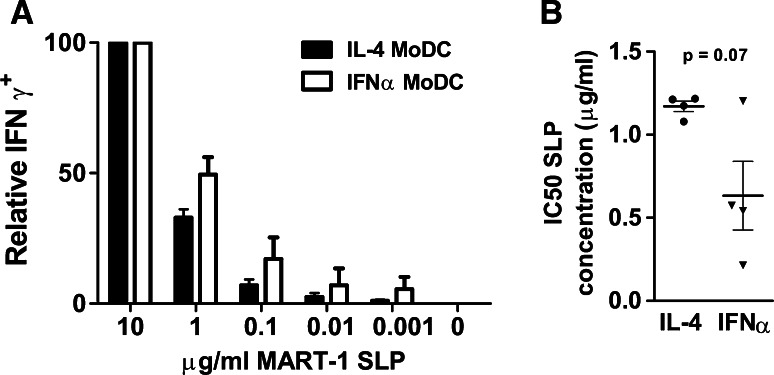
Cross-presentation of a 25-mer synthetic long peptide (SLP) by MoDC. **a** Immature IL-4 (*black bars*) or IFNα MoDC (*white bars*) were loaded with a titration of MART-1: melanoma-associated antigen recognized by T cells 1 (MART-1) 25-mer SLP (aa16-40L). After loading and maturing overnight, MoDC were co-cultured with a MART-1 (aa26-35)-specific CD8^+^ T cell line, and intracellular IFNγ was quantified. Reactivity data at the highest MART-1 SLP concentration (10 µg/ml) were set to 100 % and were, respectively, 53 and 41 % of the total CD8^+^ MART-1 T cells after co-culture with IL-4 or IFNα MoDC (*n* = 4). **b** The corresponding cross-presentation efficiency plotted as the SLP concentration, at which 50 % of the CD8^+^ T cells produced IFNγ (IC50), where the percentage of IFNγ producing CD8^+^ T cells at 10 μg/ml was set to 100 % (*n* = 4). Statistical significance was determined using a Student’s *t* test

We next determined the capacity of MoDC to cross-present a 25-mer MART-1 SLP (aa16-40L) and MART-1 from blebs. To this end, immature HLA-A2^+^ MoDC were loaded overnight with either blebs (derived from a stably MART-1-expressing AML HL60 cell line) or with the MART-1 SLP, and maturation was induced 1 h post-loading. Loaded and matured MoDC were subsequently co-cultured for 5 h with a CD8^+^ cytotoxic T cell line, recognizing the MART-1_aa26-35_ immunodominant epitope in an HLA-A2 restricted fashion (MART-1 CTL), after which the percentage of IFNγ producing MART-1 CTL was determined by intracellular staining using flow cytometry. Unfortunately, this methodology proved to lack the sensitivity to assess cross-presentation efficiency of the MART-1_aa26-35_ epitope from blebs. While both IL-4 and IFNα MoDC were capable of cross-presenting the MART-1_aa26-35_ epitope from the MART-1 SLP, cross-presentation by IFNα MoDC was more efficient (Fig. 3a). The corresponding mean cross-presentation efficiency, depicted as the IC50 value of the maximal IFNγ production by the MART-1 CTL line (Fig. 3b), was 1.17 μg/ml for IL-4 as compared to 0.63 μg/ml for IFNα MoDC (*p* = 0.07). These data demonstrate a clear trend toward a more efficient cross-presenting capacity of IFNα MoDC for soluble protein, in line with previous reports [13, 14].

### IFNα and IL-4 MoDC have similar capacities to prime antigen-specific CD8^+^ T cells in vitro

**Fig. 4:**
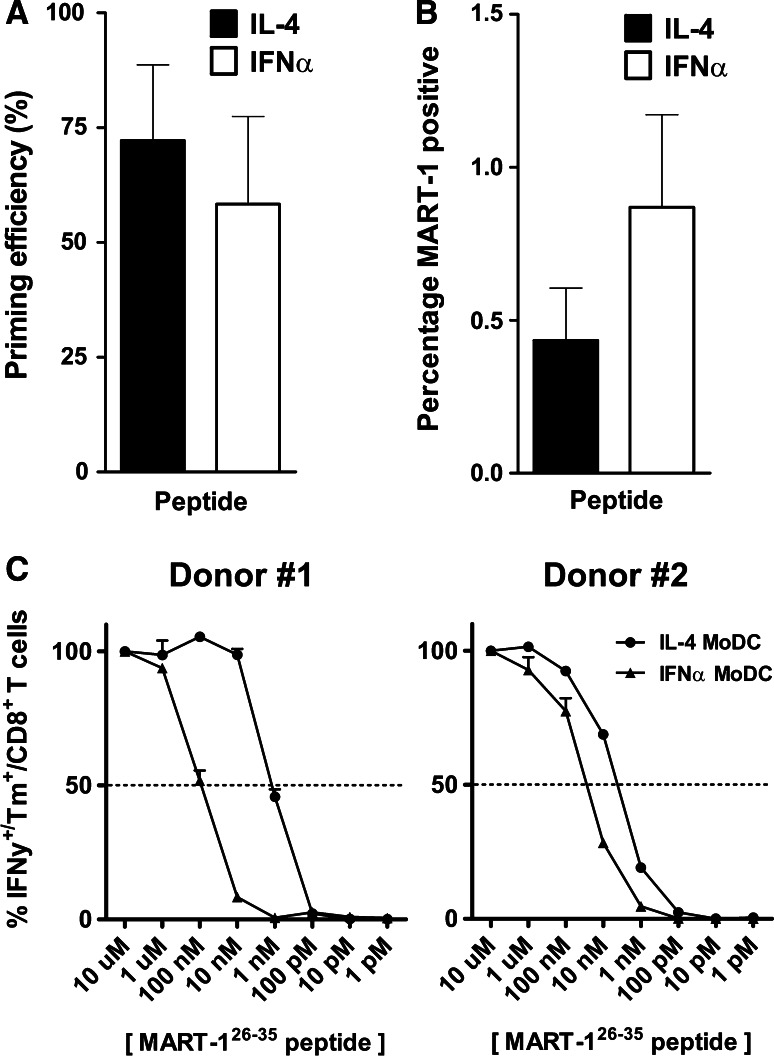
MoDC priming efficiency and primed CD8^+^ T cell avidity. **a** The priming efficiency of six healthy donors was determined by analyzing the number of wells that were scored positive over time, as a percentage of the initiated number of wells, for both IL-4 (*black bars*) and IFNα MoDC (*white bars*; *n* = 36). **b** The percentages of MART-1 dextramer-positive CD8^+^ T cells per well, averaged over six donors. Statistical significance was determined using a Wilcoxon matched-pairs signed-rank test. **c** MART-1 dextramer-positive cells were sorted and expanded, after which their avidity was analyzed. Shown are the avidity assays of two donors in which we were able to prime, sort, and expand MART-1 dextramer-positive CD8^+^ T cells to sufficient numbers (*n* = 2)

We next assessed the ability of IFNα versus IL-4 MoDC to prime MART-1_aa26-35_-specific CD8^+^ T cells. MoDC were loaded exogenously for 2 h with the 9-mer MART-1_aa26-35L_ peptide, after which CD8^+^ T cells were primed and weekly restimulated with peptide-loaded IFNα and IL-4 MoDC, to analyze antigen-specific priming capacity in an autologous setting. From six healthy donors (six parallel wells per donor), we observed MART-1-specific CD8^+^ T cell expansion by specific HLA-A2 dextramer staining in 26/36 wells (72 %) and 21/36 wells (58 %) for peptide-loaded IL-4 MoDC versus IFNα MoDC, respectively (Fig. 4a). In terms of percentage of CD8^+^ T cells staining dextramer positive, the average percentage per positive well (>0.1 % dextramer staining) was higher in the IFNα MoDC versus IL-4 MoDC co-cultures (mean 0.87 vs. 0.43 % respectively, Fig. 4b). Due to high variability, neither of these differences reached statistical significance.

We managed to sort, expand, and test the avidity of IL-4 versus IFNα MoDC-primed MART-1 dextramer-positive CD8^+^ T cells in two out of six donors (Fig. 4c), revealing a higher functional avidity of the IL-4 MoDC-primed T cells in both instances (half max. IFNγ production at a mean of 1.9 vs. 57.1 nM peptide for IL-4 and IFNα MoDC-primed T cells, respectively).

### More efficient cross-priming of antigen-specific CD8^+^ T cells by bleb-loaded IL-4 MoDC

**Fig. 5:**
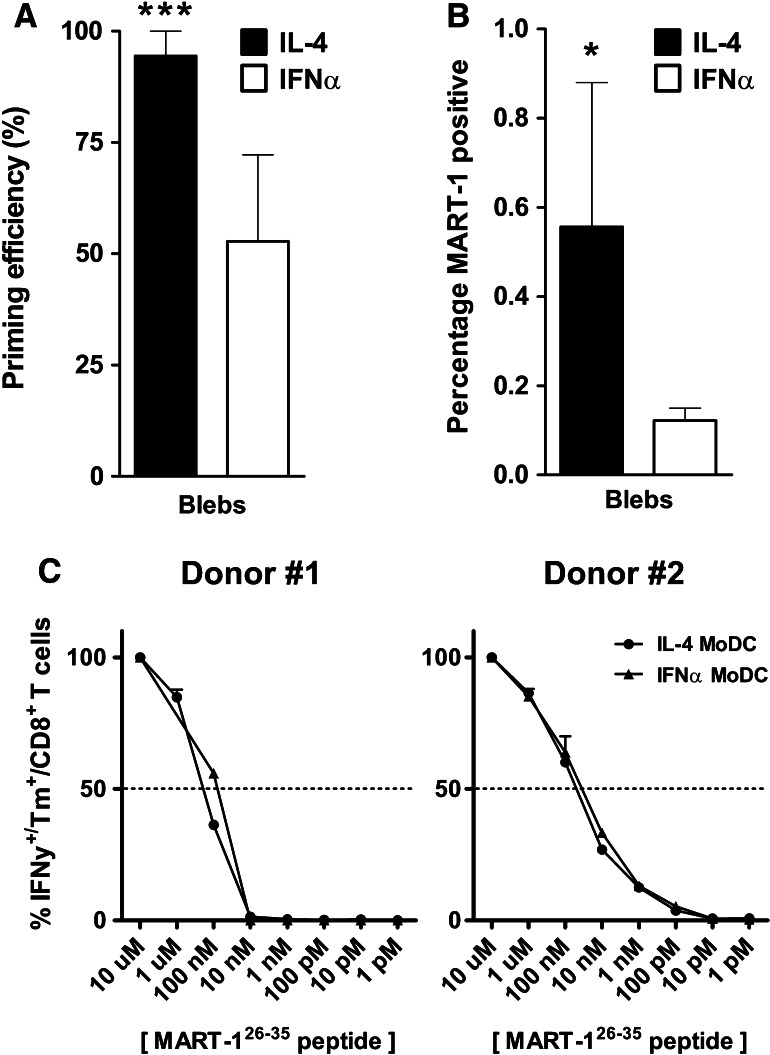
Cross-priming efficiency of bleb-loaded MoDC and resulting CD8^+^ T cell avidity. **a** The priming efficiency in six healthy donors was determined by analyzing the number of wells that were scored positive over time, as a percentage of the initiated number of wells, for both IL-4 (*black bars*) or IFNα MoDC (*white bars*; *n* = 36, i.e., six parallel wells per condition per donor). ***p ≤ 0.001 Fisher’s exact test. **b** The percentages of MART-1 dextramer-positive CD8^+^ T cells per well, averaged over six donors. *p ≤ 0.05 Wilcoxon matched-pairs signed-rank test C. MART-1 dextramer-positive cells were sorted and expanded, after which their avidity was analyzed. Shown are the avidity assays of two donors in which we were able to prime, sort, and expand MART-1 dextramer-positive CD8^+^ T cells to sufficient numbers (*n* = 2)

We next analyzed the capacity of both MoDC types to cross-prime MART-1 CD8^+^ T cells, after loading and maturing the MoDC overnight with MART-1-expressing HL60 AML blebs. Both MoDC types were capable of subsequent priming of MART-1_aa26-35_-specific CD8^+^ T cells. In contrast to peptide-loaded MoDC (Fig. 4a, b), the priming efficiency (mean 94 vs. 53 %, Fig. 5a, *p* = 0.06) *and* the mean percentage of MART-1-specific dextramer-positive CD8^+^ T cells (mean 0.56 vs. 0.12 %, Fig. 5b, *p* ≤ 0.05.) was higher when CD8^+^ T cells were primed with IL-4 MoDC as compared to IFNα MoDC. However, CD8^+^ T cells primed with either MoDC type recognized their cognate antigen with a similar avidity (Fig. 5c); of note, these T cell bulks were derived from the same donors as were the bulk cultures used for avidity analysis upon priming to the 9-mer peptide (Fig. 4c).

## Discussion

The need for targeting multiple TAA or preferably the utilization of an individual’s unique mutanome for inducing tumor-directed immunity in vaccination strategies, has become evident in recent years [19, 20]. Tumor-derived “whole” cellular vaccines have therefore regained interest, especially with the recent insights in immunogenic cell death [21]. We have previously reported on the beneficial effects of using apoptotic AML-derived blebs as a source of TAA for in situ loading of skin DC [11], as well as MoDC loading in vitro [6]. As MoDC cultured in the presence of GM-CSF and IFNα have been described to have a higher capacity for the cross-presentation of antigens [13, 14, 22, 23], they might constitute a more potent platform for the delivery of blebs in the context of DC vaccination strategies. We therefore conducted a head-to-head comparison of these MoDC subtypes.

The expression levels of cell surface antigens found were similar to those described in literature [12, 13], and importantly, loading of blebs did not interfere with maturation of either MoDC type.

Our data clearly show that IL-4 MoDC are superior in taking up exogenous antigen, as the uptake of apoptotic blebs, pinocytosis, and receptor-mediated endocytosis were significantly increased, as compared to IFNα MoDC. This difference could not be related to differential expression of receptors previously reported to be involved in the uptake of apoptotic fragments, e.g., LOX-1, which we were unable to detect. The observed lack of LOX-1 expression on IFNα (as well as IL-4) MoDC as compared to earlier studies might be explained by the IFN α-subunit and/or source used for differentiation. Whether this also explains the observation that IL-4 MoDC were more potent in ingestion apoptotic blebs remains unclear, since this difference might also be explained by the used antigen source: apoptotic blebs instead of apoptotic cells. Additionally, the modalities used to induce MoDC maturation differ between the conducted studies and ours, which can result in functional differences. It will therefore be of interest to study the functional consequences of different activating stimuli on both types of MoDC in more detail. Moreover, since two types of DC are compared, some of the observed effects do not necessarily reflect a difference in IFNα MoDC, but could also reflect differences in the IL-4 MoDC. These differences in uptake did, however, not account for the observed higher priming efficiency of allogeneic CD4^+^ T cells per se, as unloaded IL-4 MoDC induced a similarly elevated proliferation rate over IFNα MoDC (data not shown). The differences in T cell proliferation by IL-4 MoDC could be a result of the more mature phenotype, in particular the increased expression levels of HLA class II, CD80, and CD83. Since we did not observe striking differences in production of cytokines that we tested (IL-1β, IL-6, IL-8, IL-10, IL-12, and TNFα) by matured MoDC, this is not likely to result in altered T cell proliferation, although we did observe more robust production of TNFα by IFNα MoDC after maturation.

Despite the more efficient uptake of soluble antigens by IL-4 MoDC, IFNα MoDC displayed a higher ability to cross-present the MART-1_aa26-35_ epitope derived from a 25-mer MART-1 SLP, similar to what was described previously for OVA [14]. Although we observed a trend toward a higher mean outgrowth percentage of MART-1-specific CD8^+^ T cells when priming with MART-1_aa26-35L_ peptide-loaded IFNα MoDC, the priming efficiency between IL-4 and IFNα MoDC was similar. However, the avidity of CD8^+^ T cells that were primed with peptide-loaded IL-4 MoDC was higher compared to that of CD8^+^ T cells primed with IFNα MoDC (mean of ~2 vs. ~57 nM, respectively). The underlying mechanism leading to the higher avidity remains unclear, but it could be the result of the more mature phenotype of IL-4 MoDC, and possibly traces of the beneficial cytokines (IL-6 and especially IL-12p70 [24]) as observed after CD40 ligation of immature IL-4 MoDC. Although the functional CD8^+^ T cell avidity between bleb-loaded IL-4 and IFNα MoDC was very similar, a higher percentage of MART-1 positivity was achieved when primed with bleb-loaded IL-4 MoDC.

In this study, we used IFNα as a differentiation-inducing cytokine, but it will be of interest to analyze the effect of adding IFNα in the maturation cytokine cocktail of IL-4 MoDC, on their cross-priming ability, since it has been shown that this has beneficial effects on CCR7 expression and T cell responses [25].

We show that both IFNα and IL-4 MoDC are efficient primers of CD8^+^ T cell responses. However, the choice of either MoDC type as vaccination vehicle depends on the antigen source used. SLP-based therapies could benefit from using IFNα MoDC, whereas IL-4 MoDC appear better suited for cross-priming of CD8^+^ T cells to epitopes derived from apoptotic blebs, most likely due to their superior bleb uptake.

## Abbreviations

ACR: Apoptotic cell remnants
AML: Acute myeloid leukemia
CFSE: Carboxyfluorescein succinimidyl ester
CR: Complement receptor
DC: Dendritic cells
HLA: Human leukocyte antigen
IFN: Interferon
IL: Interleukin
imMoDC: Immature MoDC
MART-1: Melanoma-associated antigen recognized by T cells 1
matMoDC: Mature MoDC
MLR: Mixed leukocyte reaction
MoDC: Monocyte-derived dendritic cells
PBL: Peripheral blood lymphocytes
PBMC: Peripheral blood mononuclear cells
PGE-2: Prostaglandin E-2
SLP: Synthetic long peptide
TAA: Tumor-associated antigens
T_H_: T helper
TNFα: Tumor necrosis factors
